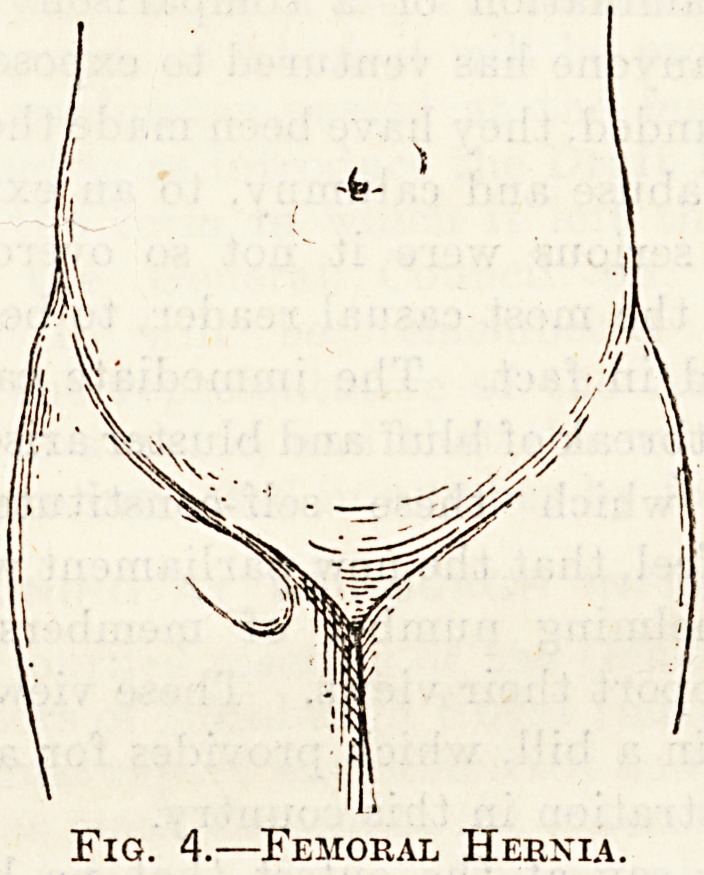# The Hospital. Nursing Section

**Published:** 1906-02-03

**Authors:** 


### The Hospital.

### Nursing Section.

Contributions for "THE HOSPITAL," should be addressed to the EDITOR, "THE HOSPITAL" NURSING SECTION, 28 & 29 Southampton Street, Strand, London, W.C.

No. 1,010.—VOL. XXXIX. SATURDAY, FEBRUARY 3, 1906.

### Queen Alexandra's Bereavement.

THE heart of every nurse in the Kingdom goes out in sympathy to Queen Alexandra in the bereavement which she has sustained by the sudden death of King Christian of Denmark. The Queen is not only President of the Royal National Pension Fund, but, as head of the Imperial Military and Naval Nursing Services and the Jubilee Institute, is directly connected with the most important nursing organisations. The personal interest which she takes in the work of the hospitals naturally leads nurses generally to regard her with their dutiful respect and affection. All nurses are animated by a keen desire that at a time like the present their condolences should be offered to her Majesty, and we have the honour, as their representative in the press, to assure her Majesty that her present grief is sincerely shared by the large class whose welfare she has done so much to promote both before and since she became the Queen Consort.

### Notes on News from the Nursing World.

### INCREASING POPULARITY OF THE MILITARY SERVICE.

WE are glad to learn that the terms of service in Queen Alexandra's Imperial Military Nursing Service, which came into force on April 19, 1904, and have therefore now had a fair trial, have had an excellent effect in attracting candidates. In the period which has since elapsed a very large number of nurses have applied; but as the standard is high, only the best are selected, and there are consequently still several vacancies for staff nurses. These, as our columns testify from time to time, are being filled up quickly. An erroneous impression has prevailed that none but hospital-trained nurses can enter the Service; but, as a matter of fact, certificates of training from the leading Poor-law infirmaries are readily accepted, providing that candidates are otherwise eligible. The question also occasionally arises whether nurses are bound for any number of years when they join the Army Service, and many, we believe, think that they are. But this is not the case. They can leave whenever they wish. The option is rarely exercised, especially when the nurses have become used to the life and are able to appreciate its advantages.

### HOSPITAL SISTERS, BEWARE!

WE are informed that letters, of which the following is a copy, have been sent to the head sister of Charing Cross, the German, Great Northern Central, Guy's, Italian, London, King's College, Metropolitan, Middlesex, North West London, St. Bartholomew's, and St. George's Hospitals. The letter is signed "E. A. Rogers," and headed "London Nurses' Institution, (Temporary Address) 186 Aldersgate Street, London, E.C., December 4, 1905."

It has been brought to my notice that no provision has yet been made for the old age of nurses in our London Hospitals, and I shall be much obliged if you would kindly let me know whether any scheme is in operation in your hospitals for the old age and care of aged nurses. I should also like to know whether you would like to be a member of the Committee of Management in order that the above institution can be brought into existence. Please forward this letter to the Head Sisters in the order mentioned on the attached list, stamps for which purpose are enclosed, so that after the letter has been received by the Head Sister of St. George's Hospital it will be despatched to myself. You will, of course, understand that the replies are enclosed in the envelope going from Head Sister to Head Sister.

From inquiries made it appears that the address is that of the Young Men's Christian Association, but Mr. Rogers is not an official of that Association, but merely an associate. It further appears that (1) "The London Nurses' Institution" is not known to the officials of the Y.M.C.A.; (2) nothing is known of Mr. Rogers beyond the fact that he is an associate; and (3) that the officials object to the Association's address being used by members for purposes of circularising. No doubt the head sisters who have received the communication in question will not trouble themselves further in the matter. They know full well that the Royal National Pension Fund for Nurses, the invested funds of which now amount to £1,000,000, is an institution which provides for the co-operation of all hospitals in a businesslike scheme for the old age and care of aged nurses.

### NURSES AND INTEMPERANCE.

A WELL-KNOWN public man in the Midlands sends us a letter on the question of "Nurses and Intemperance," which we print at his request, because we think it very desirable to remove the impression which he seems to have formed. We recently referred to the manner in which private nurses are often tempted to partake of alcohol unwisely in some families; but our correspondent, while agreeing with our comments, says that there is another side to the question. He goes on to illustrate his contention by mentioning a very sad case in which a patient, the daughter of a family of abstainers, was induced by the nurse attending her to believe that alcohol was necessary, and emerged from her illness a habitual drunkard. It is, of course, impossible to condemn too severely the conduct of any nurse who recommends a patient to take alcohol. The obligation of prescribing, or of forbidding, it devolves entirely upon the medical attendant. But we are convinced that the experience to which our correspondent alludes is as rare as it is melancholy. Even in the disastrous event of a nurse herself giving way to drinking habits, we believe that there are very few who would stoop so low as to try and persuade their patients to copy their example. It remains true that, of the two, the nurse is in greater danger of being thoughtlessly persuaded to drink a glass of wine to her detriment than the household she enters of being contaminated by her presence.

### TRAINED NURSES ON STEAMSHIPS.

We learn that trained nurses are employed in seagoing vessels by the Booth Steamship Company, Limited, whose headquarters are 20 James Street, Liverpool. The vessels ply between Liverpool and Brazil. Applicants must be over the age of thirty, good sailors, and those who have had experience in the nursing of fever cases are preferred. The work, which is distinct from that of a stewardess, is to nurse all sick persons, whether passengers or crew, under the directions of the ship's doctor. All this sounds very satisfactory, but it remains to be added that there is no vacancy, nor likely to be one, for a long time, as the Company have so many names already on the list of applicants for appointments. If, however, the Booth Steamship Company find it worth while to engage fully-trained nurses for their vessels, the owners of larger fleets should surely consider it desirable to make the experiment.

### THE EAST LONDON NURSING SOCIETY.

In view of the possibility of the East London Nursing Society being compelled, owing to lack of adequate funds, to reduce the number of their nurses, the account which a former member of the staff gives in another page, will be perused with special interest. Our contributor shows both the difficulties and the advantages of carrying on the work, and her desire that every poor part of London should be as well cared for as the particular area covered by the East London Nursing Society, is yet another reason for not allowing the strength of the organisation to be diminished, which must surely appeal to many. We are glad to learn that the nurses employed by the Society are the recipients of many little kindnesses from the subscribers, and that recreations are permitted to occupy a portion of their busy life.

### TRADES' COUNCILS AND NURSES' INTERESTS.

A sharp slap in the face has been administered to the Glasgow Trades Council, who took upon themselves to appoint a Committee to inquire into the recent dismissal of nurses at Gartloch Asylum. In consequence of the decision of the Glasgow Parish Council, condoning the action of the Medical Superintendent at Gartloch, the Trades Council deemed it advisable to consult the dismissed nurses before taking any further steps in the matter. The reply was that "the nurses did not consider that it could be to their interest to pursue the agitation." Precisely; and we hope that the Glasgow and other Trade Councils will in future refrain from interference in issues which are outside the range of their legitimate jurisdiction.

### MATRONS AND INTENDING PROBATIONERS.

It is very necessary that Matrons of hospitals should see candidates for probationerships, but we also think that the candidates should not be asked to travel a long distance, at their own expense, unless there is a reasonable chance of their engagement; and in order to minimise risks the conditions of engagement should be clearly set forth before the journey is undertaken. A correspondent complains that after she had sent the names of her referees to the Matron of a hospital at a long distance, and they had filled in the forms forwarded to them, the Matron informed her that a personal interview was essential. Our correspondent says that at the outset the Matron, in replying to her, intimated that, though in the advertisement for probationers it was stated that premiums were required, they were being abolished, and the applicant would not be expected to pay one; but that on her arrival at the hospital the Matron asked her if she was willing to pay a premium. She again said that she was not able to do so, and other questions having been put to her, including one as to whether she could come before Christmas if sent for, she returned home. A day or two later she received a letter to the effect that she could not be accepted as a probationer. There may have been good reasons for this decision; but it seems hard on the applicant that she was not explicitly told when she applied in the first instance that a premium and a personal interview were both indispensable.

### MIDWIFERY TRAINING.

A case of great interest to intending midwives was heard at the Brompton County Court on Friday last. The plaintiff went to a professional midwife to receive training for her midwifery certificate, and paid the sum of 24 guineas, for which a receipt was given "for training." Subsequently, as the plaintiff stated, she ascertained that the midwife was not qualified to grant her a certificate, and she therefore sued her for the recovery of the money she had paid. The defendant, however, affirmed that she was certificated, having been in practice before the Act was passed, and that while she knew that she could not sign a certificate herself, she told the plaintiff before the money was paid that she must attend 20 cases, which she had not done. The Judge, accepting the defendant's statement as accurate, found for her, but without costs. The moral of this case is that training in midwifery should be sought at the schools which are recognised by the Central Midwives Board.

### COMMON SENSE IN THE NURSING OF HOMICIDAL PATIENTS.

It is impossible to exaggerate the importance of common sense and attention to practical trifles in the nursing of the insane. There was an inquiry the other day respecting the death of a man of independent means at a mental asylum in Clapton, and the evidence proved that it was partly due to the patient swallowing pieces of the glass from a framed calendar which had been broken. He had previously managed to swallow a circular piece of metal. The Medical Superintendent admitted that it would have been "wiser not to have left the glazed calendar in the room." Glazed calendars, or glass in any form, should never be within the reach of a patient suffering from homicidal mania; if it is there the temptation to try and use it for the purpose of self-injury is sometimes irresistible.

### THE DIETARY OF POOR-LAW NURSES.

The importance of Mr. Baldwyn Fleming's report which touches on the dietary of indoor officers in Poor-law institutions is generally recognised, and it has elicited the opinion that the abolition of the weekly fixed dietary will never be secured by representation to Boards of Guardians. We think that this is not quite fair. There are Boards of Guardians who have already conceded to the officers the liberty of varying their diet to suit their fancy, and the superintendent nurses of several well-known Poor-law infirmaries have testified, through our columns, to the advantages of the concession. But there is no doubt that direct intervention on the part of the Local Government Board would be the most rapid, and the most satisfactory method of dealing with the matter. We cannot too strongly insist that frequent change of food is needed, not merely because the nurses get tired of the same fare, but also in the interests of health; and it is, of course, essential that the quantity should be sufficient. The complaints of insufficient rations are, however, much fewer, so far as nurses are concerned, than those of want of variety.

### CORK'S SOLITARY DISTRICT NURSE.

It is now five years since the Cork District Nursing Association was formed and a nurse engaged to look after the sick poor of the town. And although Londonderry, a far smaller town than Cork, now possesses five district nurses who have ample work to do with ample funds forthcoming to support them, Cork, with a percentage of consumption higher than any city in the Kingdom, still continues with its lonely nurse, who pays fewer visits than she would like to do because so often she is never told when the poor are ill, and whose expenses, modest though they be, are barely covered by the subscriptions to the Association. This is not the fault of the nurse, and at the last annual meeting testimony was given by those who know all the ins and outs of the work done by her, that it is as excellent as that of any of her predecessors. Yet the fact remains that so far the Association has taken no real hold on the people of Cork. A clergyman in great distress over a sick parishioner, who was asked why he did not send for the Jubilee nurse, admitted that he never even thought of doing so; and though there are numerous rich merchants in the city who could pay all the yearly expenses of the nurse and not feel the loss of the money, the lady collectors all asserted that they had great difficulty in collecting the insignificant sum of £93 9s. 4d., which left the Committee with a deficit of £4 13s. 4d. on the year's working.

### REGISTRATION AND REPRESENTATION.

On Wednesday next a special general meeting of the Corporation of the Royal British Nurses' Association will be held at 11 Chandos Street, Cavendish Square, at which it will be proposed to rescind the resolutions passed at the meeting on January 7, and to re-introduce the Draft Registration Bill in the form in which it left the special meeting of the General Council on Monday, January 8. It will be remembered that on January 17 the representative of the Association was limited to a nurse, and that the nurse representatives were increased in number by four.

### THE TRAINING AT EDINBURGH INFIRMARY.

At the adjourned meeting of the General Court of Contributors of Edinburgh Royal Infirmary last week the annual report was submitted and adopted. Reference was made therein to the extension of the period of the training of probationers, which up to the end of 1903 had been for two years only, and it appears that all the agreements for the original time have been completed, and that the three years' term has now come into full operation. The change was first discussed by the managers in 1902, and finally decided upon, after the most careful consideration, in July 1903. They are not likely to regret the fact that they have brought the Edinburgh Infirmary into line with the other great nurse-training schools.

### LECTURES ON MIDWIFERY.

The next course of lectures at the Incorporated Midwives Institute, 12 Buckingham Street, Strand, in preparation for the Central Midwives Board examination, commences on February 20. The lectures on midwifery are given by teachers approved by the Board; lectures on monthly nursing and house sanitation are included in the course, and coaching classes are held by an experienced teacher. At the end of the course the Institute holds an independent examination and issues a certificate to successful candidates.

### SHORT ITEMS.

Mrs. Palmer, author of "Lessons on Massage," who has been in Canada and the United States since last spring, visiting later many of the Western sanatoria, will return to London this month in order to commence her Spring classes on April 2.

### The Nursing Outlook.

"From magnanimity, all fears above;
From nobler recompense, above applause,
Which owes to man's short outlook all its charm."

### BLUFF AND BLUSTER.

Some astonishment and more confusion has been created in regard to nursing matters by "the stage army" leaders, from time to time, in the past Much has been said in the newspapers of late as to the mental aberration caused to many people as one direct result of the excitement due to the recent elections. It would seem that "the stage army" leaders must have been caught by some such fever, if their state of mind may be gathered from their utterances of late. The policy pursued for years has been to make statements which will not bear the test of examination or a comparison with sober facts. If anyone has ventured to expose the fallacies propounded, they have been made the objects of persistent abuse and calumny, to an extent which would be serious were it not so overdone as to appear, to the most casual reader, to be ridiculous and absurd in fact. The immediate cause of the present outbreak of bluff and bluster arises from the certainty, which these self-constituted leaders appear to feel, that the new parliament will contain an overwhelming number of members who will readily support their views. These views are to be embodied in a bill, which provides for a system of nurse registration in this country.

We may say at the outset that no bill is ever likely to become law which does not fairly represent all the interests concerned. A democratic parliament, like that which will shortly sit at Westminster, is not likely to favour fancy franchises to the exclusion of representative control in nursing or any other affairs. Yet the "stage army" leaders have so drafted their bill as to ignore the more important interests which have borne the cost and burden of training nurses in the past. But such interests must naturally and properly have a paramount voice in any legislation affecting nurses as a whole. The idea seems to be to assume, that the fact that a woman has qualified as a trained nurse, may be taken as evidence, that she does not know her own mind, or maintain the educated intelligence which her training and experience should certainly have given her.

A great stir is raised, for instance, in regard to the direct representation of nurses on any General Council charged with the duty of supervising the registration and education of nurses in the United Kingdom. The insincerity of this movement becomes, however, apparent, when the proposals are examined in detail. Everyone, who has a knowledge of the subject, realises that very few nurses, who have to earn their living by their profession, can in fact, devote the time which will be necessary in order to become efficient members of any General Nursing Council, which must meet frequently and continuously during many days in each year, if it is ever to adequately discharge its duties. It is further realised by the more intelligent nurses, that very few of their number have had the experience, or possess the knowledge, to enable them to deal efficiently with many questions on which such a council will have to lay down regulations and enforce discipline. It is no doubt essential that the general body of nurses shall be directly represented by those, who will be in close touch with their requirements and wishes, but it must not be forgotten that the whole body of the medical profession has but five direct representatives on the General Medical Council. It should be remembered, too, that if the General Nursing Council is made so large as to be unwieldy it will become unworkable in practice. The problem of securing adequate nurse representation, though important, is not a matter to be treated in a controversial spirit, such as that the "stage army" leaders are setting themselves to arouse.

The actual number of trained and certificated nurses, to-day, does not in all probability largely exceed the number of registered medical practitioners. The question is, therefore, a simple one, and can be quickly settled on adequate lines without heat or difficulty. Those who wish to solve it wisely, in the best interests of all concerned, should display sobriety and statesmanship. Certainly bluff and bluster are methods which will not appeal to the great body of trained nurses in this country, who recognise that they are calculated to injure and not to aid the cause of the nurses, whilst they are sure to destroy any weight which might otherwise attach to the utterances of those who employ them.

Neither Parliament nor the public will lend their aid in support of a party which proves by its conduct to be out of sympathy with current nursing opinion. We hope therefore that the bluff and bluster party will mend its ways. It is too large a presumption, we may, in any case, remind the "stage army," to continue the farce of permitting its leaders to pose as independent and separate interests, by merely calling themselves the Society for State Registration, the Matrons' Council, the Registered Nurses' Society, or any other names, however numerous, they may choose to take. The truth is, neither the public nor the profession will longer be humbugged into the belief that these so-called leaders are in fact anything but a few unhappy spirits, who cannot cease their struggles to obtain the control of nursing affairs in this country. Yet these struggles must prove hopeless, and the "stage army" organisers had far better go quietly to sleep, and henceforward rest from troubling themselves or others further in nursing matters.

### Abdominal Surgery.

By HAROLD BURROWS, M.B., F.R.C.S., Assistant Surgeon to the Seamen's Hospital, Greenwich, and to the Bolingbroke Hospital, Wandsworth Common.

### HERNIA.

A HERNIA, or "rupture" as it is often called, is a sac of peritoneum which protrudes through the deeper layers of the abdominal wall, and which may contain a portion of the great omentum or intestine or other abdominal organ. Unless it is very small a hernia usually can be detected by inspection. When the "hernia is down," as the expression is, that is to say, when the peritoneal sac is not empty, the hernia appears as a lump close under the skin and superficial tissues; and when the patient coughs a momentary expansion of the lump can be observed. This impulse on coughing is nearly characteristic of hernia, and serves to distinguish it from other swellings such as enlarged inguinal glands; but in this connection it must be remembered that when a hernia becomes strangulated the impulse on coughing no longer appears.

Another feature of hernia is that the lump is not always present; it comes and goes. In some cases it "comes down" at rare intervals only; in others, unless restrained, it appears whenever the patient is up and about.

A general impression of the anatomy of hernia may be gathered from fig. 3. It will be seen that the coverings of the hernial sac are the skin and subcutaneous tissues, and the thinned out muscular and fascial layers of the abdomen. The narrowest part of the sac usually is at the spot where it communicates with the abdominal cavity, and this constricted part is known as the neck of the sac. It is at

FIG. 3.—DIAGRAM OF A HERNIA.

*s*, skin and superficial fascia; *m*. muscular and aponeurotic layer; *p*, peritoneum; *n*, neck of the sac.

this point that the contents of a hernia are most liable to become strangulated.

### CAUSES.

There are two factors in the causation of hernia. In the first place there must be some weak spot or deficiency in the abdominal wall which allows the peritoneum to bulge through, and in the second place, the pressure within the abdomen must be sufficiently high to force the peritoneum through the weak spot. The intra-abdominal pressure is increased when the body is in the erect position; it is also increased by distension of the intestines, and by strong expulsive efforts as in crying, coughing, urinating and straining at stool; and any of these will tend to force the abdominal contents into any hernial sac which is present. This matter is of importance in connection with treatment.

With regard to the other factor—the weakness of the abdominal wall—there are certain places where the abdominal walls are naturally weak, and where, on this account, hernia is most apt to occur.

### VARIETIES OF HERNIA.

These weak places are the inguinal, the femoral, and the umbilical regions, and consequently in-

FIG. 4.—FEMORAL HERNIA.

guinal hernia, femoral hernia, and umbilical hernia are the three commonest varieties, although there are other less common forms.

In addition, any part of the abdominal wall may be so weakened by injury that hernia develops. This is most commonly seen as a result of operations on the abdomen; and the resulting protrusion is known as a traumatic or post-operative hernia.

The following tabular classification may be useful for reference:—

1. Umbilical.

   (*a*) In infants.

   (*b*) In elderly patients.

2. Inguinal.

   (*a*) Congenital.

   (*b*) Acquired.

3. Femoral.

4. Other forms.

   (*a*) Traumatic and post-operative.

   (*b*) Epigastric, obturator, lumbar, diaphragmatic, etc.

*Umbilical hernia* is most commonly seen in infants and in elderly women. Two forms are met with in babies. In the first the hernia is present when the child is born. In this rather rare form the umbilical cord is dilated at its junction with the abdomen, and coils of intestine occupy the dilated part. But the other and usual form of umbilical hernia, which is present in a considerable percentage of all children, does not exist at the time of birth. It forms a short while afterwards, and is due to yielding of the umbilical scar. The protrusion is small and as a rule undergoes spontaneous cure, although occasionally it increases in size and causes considerable trouble.

In elderly people umbilical hernia is most frequently seen in women who have borne many children.

*Inguinal hernia* appears as a lump in the groin, just above the inner half of Poupart's ligament. When large it may extend downwards into the scrotum in the male, or into the labium majus in the female. The great frequency of hernia in the inguinal region is due mainly to the anatomical fact that in babies there exists a canal which communicates above with the peritoneal cavity and which passes downwards to the scrotum or the labium majus, and is known as the *processus vaginalis testis* in the former case, or the canal of Nuck in the latter. Naturally, the canal gradually becomes occluded, but not always so; and when it remains patent, some intestine may slip through the opening and a so-called congenital hernia is produced. (Perhaps it is worth while to explain that the term congenital, as applied to inguinal hernias, and to the majority of infantile umbilical hernias, is a misnomer, for, although the predisposition is congenital, the hernia itself is not present in these cases at the time of birth; it develops later.) Even if the canal does become occluded, a weak spot may remain where the opening has been, and an acquired hernia may form at this place. Moreover, the vas deferens and vessels of the testis in the male, and the round ligament in the female, pass out of the abdomen in this region along the inguinal canal, and the place where they emerge from the abdomen is a weak spot in the abdominal wall which may allow a hernia to develop in the later years of life.

In infants and young children inguinal hernia is nearly always due to patency of the processus vaginalis testis or the canal of Nuck.

*Femoral* hernia, like the inguinal variety, appears in the region of the groin, but there is this difference, that while the latter emerges from the abdomen above Poupart's ligament, a femoral hernia passes out below this stricture. (See fig. 4.)

The condition is rare in young children, and becomes increasingly frequent as age advances. It is more common in women than in men.

(*To be continued.*)

### The Nurses' Clinic.

### THE TREATMENT OF OPHTHALMIA.

A VERY important branch of nursing is the treatment of eye diseases, and to be proficient in this work it is necessary to spend at least 12 months in an ophthalmic hospital in order to be able to recognise the appearance and symptoms of different diseases of the eye, and to learn how to carry out different treatments ordered; also how to make the necessary preparations for operations.

It is quite essential for a nurse to have a general training before taking up this special work, as with many eye diseases there is a general constitutional disturbance as well, in which a general knowledge of training is necessary. It is almost superfluous to state that absolute cleanliness on the part of the nurse is most important for her own safety, as well as for the welfare of her patient.

Many eye diseases are highly contagious, and by carelessness can be transmitted from one eye to another. There is nearly always some discharge in contagious eye diseases, which can be easily carried to a healthy eye if the hands of the nurse are not scrupulously clean; and any basins, etc., used must be well sterilised before being used for another patient.

The principal contagious diseases of the eye are conjunctivitis, ophthalmia, neonatorum, gonorrhœal and diphtheritic ophthalmia, and granulated lids; with the exception of the latter, these varieties are all highly contagious. Great care must be used by the nurse to keep towels, handkerchiefs, etc., used by an ophthalmic patient for that patient alone.

With regard to antiseptics for the eyes, it must be remembered that strong solutions cannot be used, as the eye is such a very delicate organ. Many doctors prefer not to use antiseptics, and merely keep the eye as clean as possible by bathing frequently with sterilised water or weak boric lotion. In the case of an operation the eye would be protected with aseptic dressings.

The following are the general rules for nursing diseases of the eye, though many doctors have their favourite methods, in which case they would give their instructions to the nurse; but when it is difficult or impossible to get medical assistance these rules may be safely followed.

In slight cases of conjunctivitis the patient will often be able to bathe his own eyes frequently with warm boric solution (a teaspoonful of boric powder to a pint of water); this should be done every half-hour if necessary, and is the principal part of the treatment.

Poultices are never used for contagious diseases of the eye, as they retain the discharge and sometimes injure the delicate covering of the eye itself. If heat must be applied, let it be done by frequent bathing or hot compresses, which are applied as follows: Have a vessel of hot water by the bedside, heated by a spirit lamp if possible, or frequently changed from a boiling kettle on the fire; change the swabs on the eye about every two minutes. This should not be continued for more than half an hour at a time.

The cleaning of an inflamed eye is very difficult to do gently and thoroughly, and rough usage would cause the patient much needless suffering. This is one of the principal reasons why a nurse taking up eye work needs continual practice to be perfect, though some nurses never acquire a light, firm touch, while others will fall into it quickly. To cleanse an eye the nurse must have surgically clean hands, and protect her eyes with glasses. All the nursing solutions, basins, cotton-wool, etc., must be ready and handy for her, and placed where she will get a good light.

If the patient is a child, it must be wrapped in a sheet, with the hands inside, so that it cannot interfere with the nurse. Two people are necessary for this—one to hold the child on her lap, and the other to attend to the eyes. The one who attends to the eyes must hold the child's head backwards, between her knees. The lower lid is gently pulled down by the thumb resting on the cheek under the eye, showing the inner surface. This is washed clean by

**THE NURSES' CLINIC—*Continued.***

water dropped from a swab of cotton-wool, the stream being continued till all the pus is removed. To wash the upper lid, take the eyelashes of the lid, and pulling the lid forward, away from the eye, the thumb of the other hand is placed on the outside of the lid, which is then turned back over the thumb, the patient looking down all the time. Squeeze water in the same way as before on the lid and between the lid and eyeball till all pus is removed.

Sometimes the eyelids are so swollen that they cannot be turned, and then sometimes the surgeon snips the lids at the outer corner, when they will easily turn. This slight operation is called canthotomy, and often gives great relief by removing pressure from the eyeball and relieving the congestion.

The next item of treatment is cold application. Have a good-sized piece of ice close to the bed, and put several squares of moistened cotton-wool or lint on it; leave them on the ice till quite cold, and then place on the eyes for a couple of minutes; then change them, replacing the warmed ones on the ice, providing they are not touched by any discharge. Continue this for 20 or 30 minutes at a time, as often as ordered. As a rule this is all the treatment that comes within the province of the nurse, as the surgeon generally attends to the applications of medicines, though this may be sometimes left to the nurse.

If it is an application of nitrate of silver a piece of old linen or lint is rolled on a holder, and then the lids are drawn down or turned as in cleaning the eye, and the nitrate of silver gently rubbed on the inner surface of the upper and under lid. Sometimes after the nitrate of silver the eyelid is washed with a solution of salt and water to check the effect of the silver.

Eye patients should be kept in darkened rooms, on light diet, and the bowels kept well open. All soiled dressings should be immediately burnt if possible, and the nurse should always have a basin with disinfectant at hand to dip her fingers into.

In gonorrhœal ophthalmia—a terrible disease which causes a very large percentage of blindness in adults and children—it is most important to try and protect the sound eye if only one is affected. The best means of doing this is to apply a Buller shield, which is made as follows: Obtain an ordinary watch-glass, paste a piece of adhesive plaster to the inside, and one to the outside of the glass, leaving a hole an inch square in the middle of the plaster. The smaller piece of plaster should be on the hollow side of the glass. The outer piece, being larger than the inner one, sticks to the face above the eye, over the nose, and below the eye, the side towards the ear being left unstuck to ventilate the eye and prevent the glass from being dimmed, thereby preventing the doctor from inspecting the eye and the patient from seeing. With small children a pad and roller bandage is better, as it is not so easy to pull off. Eye nursing requires the greatest care and attention to the doctor's orders, as carelessness on the part of a nurse may result in a patient being rendered blind and helpless for the rest of his life.

### Incidents in a Nurse's Life.

### SISTER'S TEA PARTY.

There was an air of suppressed excitement in the ward. This was 3 p.m., but as 4.30 approached the pent-up feeling burst forth, and the news swept round "'Ere I say, d'you know we're to go and 'ave tea wiff Sister to-day?" Sister had promised to entertain five small guests, if they could promise to produce reports of excellent behaviour for at least one hour beforehand; this being accomplished, they were to be ready for the much-loved cake at 4.30. At the suggestion of one agile young person, aged six years, another young person of similar qualifications took upon himself the right to issue indiscriminate invitations, from which were afterwards selected discriminate guests.

There are as many ways of showing delight as there are methods of hailing an omnibus, and when all the recipients had squealed or squeaked and made other audible signs of assuring the whole ward of their speedy transplantation to Sister's room, the work of collection began. Needless to say, there was a previous "tidying" process on nurses' part, as the newly-brushed hair and shiny faces proclaimed. It is surprising how many small people can find room on one small rug. From the woolly hearthrug sprouted three smiling imps, and in close proximity were the two other imps who, owing to their non-attachment to splints, managed to produce themselves on the stroke of 4.30 with surprising independence. It did Sister good to see them all so merry, for, in spite of bandages and tenacious splints, these little patients are generally as happy as the day is long; they feel, though perhaps unconsciously, the influence of those who tend them because of their love for the little ones. Children are always at their best in their own society, and so, under the stimulus of sweet milky tea and cake, the little tongues began to waggle freely. Two friends had joined Sister, also of similar degree, who, for the time being freed from duties, were ready to enjoy the happiness of the party. The small patients soon discovered that Sister's friends also liked small boys, and the most intimate friendships began to flourish and grow as the capacity for cake grew less. "Now won't you tell me your name?" The small devotee of cake thus addressed forgot to be proper and to present for inspection the gift of his godparents, and blurted forth instead, "Sister calls me 'Dumpling' cos I's fat," and further, at the smallest instigation from its originator, "Yus, Sugar-plum dumpling when I's good and Suet-dumpling when I's naughty."

Encouraged by the approval and applause of the hostesses, the next imp became communicative, and supplied the information—in a tone which implied the expectation of an equal burst of approval—that he was "Ikey." Having gained his point and subsided, the next person called upon to declare his identity said he was known as "Sammy," whereupon shouts from the "Ikey" direction were speedily augmented by the rest, and the street song, "Sammy—you are my Sammy," was promptly in full swing, to the intense enjoyment of the perpetrator. The other two who were still to give their distinctive appellations withdrew into the shadow, for "Teddie" was slightly commonplace, whilst "Patsy" was not sure whether his real name, "Patrick," would not have been rather more befitting to the occasion.

The next appearance of edible substance was a plate of sweets—made by Sister, and quite harmless. Then came the crowning point of all, some lovely shiny medals hanging from the most patriotic of ribbons. These were pinned by one of their new friends to each palpitating little pinafore, and soon after the party broke up and left sister's room very dull and very crumby.

"What were they for, doctor?" said "the Dumpling," repeating that gentleman's question apropos of the medals, next day. "Sister said for dood tonduc." He had by this time collected two others from their generous recipients, and marched along disporting them on his plump little chest, to the admiration of all the "daddies" in the ward.

No matter if they were only reminiscences of the Coronation; the children's pleasure was just as keen as though they had been struck for the occasion.

### District Nursing in East London.

BY A FORMER MEMBER OF THE STAFF.

When I obtained a post as district nurse under the East London Nursing Society, I considered myself especially fortunate, for I had long wanted some East End experience. I found that I was installed in the central division, but lack of opportunity had prevented me inspecting my quarters before the date on which I was actually to begin work, and that eventful day happened to be Boxing Day. I do not think I shall ever forget it, the rain that was falling, the dark, dingy streets, and the general air of dirt and squalor which seemed to overpower anything else. Had Matron not been with me, I think I should have turned coward and run home again. But from the first day to the last Matron was my true friend, and up to the present day I still retain my belief that she was one of the best women that ever walked God's earth—a true, brave, sincere gentlewoman.

### A Depressing Reception.

I found I was to live at Church House. Nobody appeared to be at home but a charwoman, and the place looked very cold and wretched. My room was all "upside down," for, as the ancient dame expressed it, she was "all of a muddle," and "no nusses wasn't expected that day." Matron suggested that she might light a fire for us, but she strongly objected, saying it wasn't her work, she hadn't been told to wait on "nusses," and much more to the same effect. After some coaxing, however, she was prevailed on to bring coals and wood, and soon we had a bright fire, which seemed to put a much more cheerful aspect on things in general. Then, cheered and encouraged by Matron, I set to work, she helping me, and very soon we had made the room look a little more tidy and homelike.

### Some of my Fellow-workers.

In the midst of our endeavours, the tea-bell rang, and we went to the dining-room, where I was introduced to Sister C., one of the visiting ladies of the parish. She was dressed in a queer sort of loose blue gown, tied at the waist with a girdle of black, ropy-looking stuff, and on her head was a huge white cap, with a sort of cape to it that covered her shoulders, whilst suspended round her neck by a black ribbon was an enormous ebony cross. I found afterwards that she was a religious enthusiast, and that her sympathies were largely with the foreign portion of the parish, which, I need hardly say, was larger than the English part. Soon after tea Matron left me, and I felt myself to be fairly installed in my new home. Next morning I was summoned to breakfast at 8 A.M., and made the acquaintance of the housekeeper and another of the parish workers. Then after breakfast I began to work in earnest.

### A Cheery Old Lady Patient.

My first visit was to a poor bed-ridden woman, who received me very enthusiastically. Dear old Mrs. R——, she was an object-lesson of patience and contentment, for as she laid in bed all she could look at was a bit of brick wall opposite. She had not seen the sky for fourteen years, was wretchedly poor, and more often than not racked with pain, and yet I never failed to get a smile and a kind word from her. It was always a pleasure to see her, and oftentimes, when feeling extra tired and depressed, she was the cheerer instead of expecting me to cheer her up. Next, following my directions, I arrived at an unmistakably Jewish household, my patient being a child aged eight, suffering from scalds. This was my very first introduction to a Jewish home, and to say I was appalled is only a mild way of expressing my mingled feelings of disgust at the dirt and wretchedness, not, however, unmixed with admiration at the picturesque beauty of the children. They all spoke English remarkably well, but here I must say *en passant* that one of the parish workers was an excellent linguist, and was always ready to accompany me to a house where I failed to make myself understood, and oftentimes I came across families of Germans and Russians who knew not a word of English.

### More Patients.

Case Number 3 proved to be of a totally different type, for, after mounting countless stairs, I found myself in a neat and clean little workman's flat, where I was greeted kindly and courteously by the whole family. There were a great many of these clean little English dwellings in my district, whose occupants were chiefly employed in a huge factory near by, which gave work to some hundreds of men. In course of time I grew to know these people very well indeed, and some of them are my sincerest friends to this day. Case Number 4, a young man with cuts on his head, proved to be not at home. I was informed that he had gone to a party the night before, and had not yet returned. The fifth case was in another Jewish household, but a great contrast to the first, for, although very poor, and almost bare of furniture, the house was scrupulously clean. My patient, a sturdy child of four, bore the distinguished name of Paul Kruger, and he started yelling loudly at the sight of the "doctor lady," as his mother called me. A sore foot was soon bound up, and in the house next door I found my sixth patient, a young English girl, ill-clad, ill-fed, and obviously over-worked, with a most horrible looking ulcer eating into her ankle, and which her mother told me was getting bigger and bigger, and which nothing would heal. This patient became a great favourite, and I may as well add here that it took me five months to heal that ulcer, and then not until I had grafted some of my own skin on to it. There were in all only eleven cases to be seen that morning, but by the time I had finished my round I was quite tired and ready for my one o'clock dinner, of which I partook with the other workers, who were most sympathetic, and anxious to hear how I had been received among the people.

### Off Time.

The interval between dinner and tea was my own to rest in, take a walk, or employ as seemed best to me. As there was always a good bit of walking to be done in my morning's round, I usually preferred to stay indoors, and on this, my first day, I had to be very busy unpacking my possessions and trying to make my room look nice. Later there was the evening's round to be done, accompanied by Matron, who was, I quickly discovered, a great favourite both with the English and the Jewish people. Then came supper and prayers, and so ended my first day as a district nurse. Now began a very bright, happy time for me, though I found that all my days were not so easy as the first one had been by a long way, for I sometimes had as many as 30 patients on my book. Some of these were disagreeable, others very nice; but as a rule the English and Jewish alike vied with one another in being profoundly grateful for the smallest service rendered them. I never had many serious cases, for the London Hospital was very handy, and the doctor and nurses there always gladly rendered any help in their power. Of night work I had very little. I occasionally sat up with a critical case, and was sometimes called out in the night; but these only came as breaks in the usual routine of work.

### DISTRICT NURSING IN EAST LONDON—*continued.*

### Writing up Cases.

There was a good bit of writing to be done, as a register had to be kept of all patients, with particulars of their various complaints, etc. Occasionally these did not tell quite all the truth, for if I was called to see some dirty urchin who had been fighting and got a broken head, I had to enter it as "scalp wounds," as being more dignified on paper! There was also a list to be kept of blankets, clothing, etc., which was lent to the patients from time to time, and of which I had the charge in a special cupboard. The register had to be taken to Matron's office once a week, and entered in her weekly report. There was also a "store" list, which was some sort of a check on any extravagance in the way of using wool and lint, which, in the case of nurses fresh from hospital wards, might otherwise prove a big item.

### The Weekly Duty I Most Disliked.

One weekly duty which devolved upon me was to attend, in company with the other workers, male and female, a sort of council meeting, presided over by the Vicar and curates, and to hand in a list of new patients, with names and addresses. This, of course, was quite in order, but I strongly objected to being questioned as to what was the matter with each individual case. In one instance (that of a woman) I replied that I didn't know, and was promptly told by the Vicar that I *ought* to know, and I couldn't be much of a nurse if I didn't; he must inform Matron, and more to the same effect, until I felt ready to rush out of the room. I resolved to speak to Matron that afternoon, but on doing so found that the reverend gentleman had been as good as his word, and forestalled me. Matron was kindness itself, and agreed with me that it was not seemly to discuss the patients' ailments in a mixed meeting and wrote to the Vicar to that effect, so that I was not questioned any more, but the other workers were questioned instead. Attached to the Church House, at the time I speak of, was a free dispensary, which was opened three times a week for the benefit of all and sundry who liked to attend it. Three very clever physicians gave their services for two hours on each of these days, and listened patiently to the many tales of woe, mostly in Yiddish or broken English. I was not supposed to assist in any way, but on one or two occasions, when a small operation had to be performed, I offered my services, which I believe were gratefully accepted.

### The Jewish Sabbath.

The first Saturday I spent in my district seemed a very strange one. To go out in the morning to find closed shops, and a Sabbatical stillness pervading the atmosphere, was a new order of things. To see the black-coated and tall-hatted Jews, with their wives and daughters in all the colours of the rainbow, marching solemnly to the Synagogue made me feel altogether as a stranger in a foreign country, and then, when after sundown all their shops were opened again, the streets became transformed into the usual hurry and bustle of Saturday night. Indoors, also, there were great preparations for the *Shabbos.* The "house," which generally meant the one room of the family, was swept and garnished, all rubbish and dirt of every description being shot out into the street. A clean white cloth was put on the table, and at sundown candles were lighted. The very poorest had two or three, and I have seen as many as twelve in various parts of the room. The Jews who keep their Sabbath in the strict orthodox way will allow no Gentile over their threshold on that day, and this made things a bit awkward for me at times, but a great many concessions were made for the "doctor lady," and I was never repulsed save on one occasion, when the door was slammed in my face by an irate old Jewess. During the time that any of their great feasts or fasts were in progress I, acting on Matron's advice, visited them as little as possible, but I have sometimes been invited in to partake of sweet cakes, passover bread or "batza," and a peculiar kind of wine, very sweet, very fiery, and most stupefying. One thing which I found very difficult to teach these "children of the Ghetto" was the desirability of fresh air and cleanliness; I am afraid my efforts made but small headway. Time-honoured customs and prejudices take a lot of uprooting, and I have in turns begged, coaxed, scolded, and threatened never to visit them any more, but all to no purpose; they have faithfully promised to do my bidding, and next day I have found things exactly the same.

### Recreations.

Every nurse was allowed from fourteen to twenty-one days' holiday in the year, generally taking a time when work was slackest, because our neighbour from the next district had the work as well as her own, and there were often little recreations given by the ladies of the committee. Theatre or concert tickets, a day's outing to Kew Gardens or to some picture gallery, winding up with dinner at a restaurant, came as great breaks in our daily life, and were always greatly appreciated. We also received many little kindnesses privately from the members of the Society, sometimes being asked to luncheon or dinner at their houses, and on the whole we met with much kindness; and I found the East End of London wonderfully healthy, and kept very well all the time. In concluding I should just like to say how I wish that every poor part of London was as well cared for as that particular area covered by the East London Nursing Society, where the very poorest have a trained nurse at their beck and call, with the loan of every appliance necessary for home nursing, besides the use of the free dispensary. Are there not hundreds and thousands of the middle-classes in London who cannot afford much, but would gladly pay what they could, for the privileges that my patients looked upon as their right?

### The Typhoid Epidemic at Basingstoke.

### NURSES IN PAPIER-MACHE HUTS.

It is now four months since there first circulated a sus picion that Basingstoke was about to be visited by an outbreak of typhoid fever. The outbreak proved more serious than was expected. Beginning on September 18, no fewer than 102 cases were notified by the 30th of the same month.

It continued with more or less severity till about the middle of October, and since then a few stray cases have been notified from time to time, bringing the total number of victims to 174. The illness in many cases was of a very severe form, and of the number afflicted 15 died. The Isolation Hospital, standing a mile from the town and built a few years back to hold 40 beds, was soon full, as also the Small-pox Hospital close by, which holds 12 beds. These proving insufficient it became necessary to provide further accommodation, and six papier-maché huts were bought, each hut large enough to hold 12 beds. They were erected, painted, equipped, and ready for occupation in four days. They were most comfortable, heated by coke-stoves and lighted by incandescent gas, and each hut was furnished with an "emergency fire-extinguisher" in case of fire—happily not required.

The only disadvantage was that in stormy weather they were not quite waterproof, but on the whole fair weather favoured us. All these huts were connected with each other by a covered roadway, thus doing away with the necessity of going outdoors.

The hut in the centre was used as a kitchen, and was fitted with a large gas-stove and sink, where dishes, etc., were washed, and also served as a room for night nurses to take their night meals.

Another hut which contained three partitions was used by nurses for sleeping accommodation, four occupying each partition; these also were heated by coke-stoves kept in night and day, and many were the uses of the stoves. They provided roast chestnuts at night, hot water to wash and hot water for tea in the morning, each nurse taking her turn at this duty. It was found essential to cover the roof of this hut with tarpaulin, as occasionally an umbrella was seen over the head of a nurse in bed. How good-humouredly the nurses overcame all little inconveniences, and worked for the welfare of the unfortunate patients is known only to those in daily contact.

It is perhaps a coincidence that of 40 patients admitted to these huts, many of them severe cases, not one death occurred and only two had relapses.

The country folks passing the hospital in conveyances were often ready to offer a ride to any nurse on the line. and some of the incidents were amusing. One old man, asking, "How be they getting on," meaning the patients, on being answered, replied, "Ah, well, now; I did hear say as how they wun-ner let the corpses go home," meaning, of course, that bodies of those who had died were not allowed to be taken home.

Another kind friend volunteered a ride to some weary nurses coming home, but great was their amazement on getting in the cart to find that it contained a huge barrel of pigwash. Nevertheless, they endured it good naturedly. On another occasion it was a question as to how to get out of a high cart in which two nurses had enjoyed a ride. It was met with a suggestion from the driver that he would take them up a back street and tilt up the cart at the back! These and many other incidents will be long remembered by people who were associated with the hospital at this time. Of the hundred patients accommodated only two now remain, and it was with much joy and gratitude that many were able to join in the united Thanksgiving Service recently held in Basingstoke parish church.

### Central Midwives Board.

A meeting of the Central Midwives Board was held on Thursday last week. There were present Dr. Champneys (Chairman), Mr. Ward Cousins, Dr. Dakin, Miss Paget, Miss Wilson, and Mr. Parker Young.

### The Duties of Inspectors.

The first business was a motion by Mr. Ward Cousins that the form of suggestions accompanying the agenda of December 14 for the guidance of the medical advisers of the local supervising authorities respecting the duties of inspectors appointed to exercise general superintendence be approved and issued to the local supervising authorities. Mr. Cousins said he felt that the greater number of the local supervising authorities needed guidance from the Board in this matter. Some of the members of the Board thought that the authorities might resent any interference, especially those who had drawn up excellent suggestions themselves. Eventually it was decided, before any further steps were taken, to consult Mr. Fordham, the representative on the Board of the County Councils, and to bring the matter up again after his opinion had been obtained.

### The Proposed Reduction of Cases.

A letter from the Clerk of the Council, transmitting copy of a resolution passed by the Guardians of the Lutterworth Union suggesting the reduction from twenty to seven of the number of cases qualifying a candidate to enter for the Board's examinations was then read. This letter had come to the Board through the hands of the Privy Council, and the Local Government Board had been approached by the Lutterworth Guardians to rectify what they considered an unwarranted hardship. Mr. Parker Young moved, and Miss Wilson seconded, that an answer should be given to the effect that the Board do not approve of the suggested reduction. This was carried.

### Resignations and Appointments.

Letters were read from Dr. W. Rivers Pollock and Dr. W. Tate resigning their positions as examiners for the London and Bristol centres respectively. A letter from Dr. A. J. Wallace was read asking the Board's opinion as to whether he was obliged to sign a certificate for a midwife of having attended, to his satisfaction, a course of instruction, when the course was taken six years ago. The Board held that it was a matter of common law, and did not come within their power to decide upon. It was certain Dr. Wallace was not responsible for the present knowledge of the woman.

### A Question of Supervision.

A letter from Dr. Eustace Hill, County Medical Officer for Durham as to the extent of the exemption conferred by Rule E 21 was then considered. Dr. Hill wished to know if his inspectors might visit Poor-law infirmaries according to that clause in the Act by which it is enjoined that general supervision shall be exercised by the Local Supervising Authority. He stated that midwives in these infirmaries also sometimes laid out the dead (prohibited in Section E of the rules). He also wished to know if the midwives could be said to be under the supervision of a duly appointed medical officer if the medical officer were not resident and not present at the labours. The Board held that the inspectors could not insist on Section E of the rules being fulfilled, and that all supervision must be only general, and considered that the medical officer was "duly appointed," even if he did not actually superintend the labours.

### Notification of Midwives' Deaths.

A letter had been received from the Registrar-General as to the failure of Registrars to notify the deaths of midwives to the Local Supervising Authority. The Registrar-General stated that the midwives had been described as wife of so and so or by some other qualification, and the only way to remedy this was by letting each Registrar have a roll of the midwives in his district. But the Secretary pointed out that as the Registrars' districts were not co-extensive with the Local Supervising Authorities' districts, the whole roll would have to be supplied to each Registrar, and the Board could not possibly do this owing to the very large number of Registrars over the kingdom. Mr. Parker Young was appointed the medical member of the Board to assist the examiners in setting the paper for the ensuing examination.

### Penal Proceedings.

On the adjourned consideration of the report of the committee on the business of the Board, it was unanimously decided to hold in future the Standing Committee the week before the Board meeting, in order to expedite business, and to circulate the minutes of the Standing Committee to all members not less than three days before the meeting of the Board. In this way country members, if unable to attend the Standing Committee, would have an opportunity to consider and, if desirable, attack at the Board meeting the considerations of the Standing Committee. Miss Wilson moved "That a committee be appointed to consider the Board's penal proceedings generally, and to report thereon." She said that she had corresponded considerably with Mr. Fordham on the subject, and they were both ot opinion that much change was needed in the preparation of the materials for the cases. A committee was accordingly formed, consisting of Dr. Champneys, Mr. Fordham, and Mr. Parker Young.

### The Cheltenham Nursing Association.

Miss Wilson then moved: "That the resolution of the Board of November 23, refusing the application of the Secretary of the Cheltenham District Nursing Association for an extension of time for signing the schedules for the February examination in the case of four nurses now being trained by the association, be rescinded; and that, in the event of the above resolution being carried, in the case of candidates for the February examination trained by the Cheltenham District Nursing Association the period of one week be substituted for the period of three weeks required by Rule C 2." The point at issue was that these four nurses were not able to enter their names for the examination at the right time, though they would have completed their course of training, solely because the Board had changed the date of the examination, and the Cheltenham Association was obliged to make bookings very early, as there was much demand for their training, and they had not foreseen any change. Miss Wilson thought it would be an act of injustice to exclude these candidates, and Mr. Parker Young was of the same opinion; while the Secretary and other members of the Board contended that it was illegal for the Board to alter its ruling, and in doing so in this case they might be inflicting injustice on others similarly situated who had suffered from the change of date. Eventually Miss Wilson's motions were carried by three votes to two.

### General Business.

The report of the Standing Committee was then taken. On receipt of a letter from Dr. W. C. S. Burney, Medical Superintendent Greenwich Union Infirmary, stating that the improvements required by the Board had been already carried out, and asking that the infirmary should be recognised as a training school, recognition was granted. In reply to a letter from the Clerk to the Paddington Guardians, asking the grounds of the Board's refusal of the application of the Guardians for the approval of their infirmary as a training school, the Secretary was requested to state that the main reason for refusal was that the building was structurally unfit.

### MIDWIVES REMOVED FROM THE ROLL.

A meeting of the Board was held on Tuesday, to consider the removal of the names of certain midwives from the roll. There were present Dr. Champneys (Chairman), Dr. Dakin, Mr. Fordham, Mrs. Latter, Miss R. Paget, Miss Wilson, and Mr. Parker Young.

Six cases were brought forward, and there were no defendants. The first was that of Hannah Howe, certified midwife, who was charged with being uncleanly in person and not wearing a dress of washable material; with not taking with her appliances or antiseptics when called to a confinement; and with not keeping a register of cases, as was required by the rules. The evidence did not seem sufficiently strong to warrant removal from the roll, and the Board accordingly censured her for not conforming to the rules, and, cautioning her as to her future conduct, wished it made clear to her that she was liable at any time to be struck off the roll.

The case against Eliza Pugh was then heard. She was charged with having been guilty of negligence while in attendance on a case on October 12, 1905, in the following respects: That the perinæum being ruptured, she did not decline to attend alone; or advise a registered medical practitioner to be sent for; that when a rigor occurred on the 16th she did not then send for a doctor; that when the doctor eventually came she stated, in answer to a question from him, that the patient was not ruptured, knowing well that she was; and that an attempt had been made by an unqualified person to stitch up the perinæum. The midwife was also charged with being habitually uncleanly in person, and generally disobeying Rules E 1 and E 2. Dr. Scott said that when he was sent for by the mother of the girl on the 17th he distinctly asked the midwife if the patient were ruptured, and she denied it, while on examination he found three stitches had been put in, two of which were very septic; and he was told that they had been put in by Mr. Dudley, a man-midwife, who had been at first called in instead of a qualified practitioner. Puerperal fever set in, due, Dr. Scott thought, to the rupture being improperly attended to. Dr. Gregg, the lady inspector of the division, gave evidence to the effect that Mrs. Pugh had herself told her that she had advised the patient to stand up, and the child was born while she was in an upright position. Hence the rupture, the existence of which the woman admitted to Dr. Gregg, though she had denied the same to Dr. Scott. Dr. Gregg also said that she was uncleanly in her person, did not understand the use of a thermometer, and only had a pair of scissors, syringe, nail-brush, and some permanganate of potash. Dr. Reid, Medical Officer of Health for Staffordshire, said that the woman had told him she tried to dissuade the mother from having Mr. Dudley, and that she did not tell Dr. Scott, when he came eventually, of the rupture, as they did not want him to know Mr. Dudley had been. In her letter of defence the woman reiterated this, and said she could not get appliances because she had no means. The Board unanimously decided to remove her from the roll.

The case of Jane Tween was then heard, on charges of negligence while in attendance on a certain woman on September 17, 1905, and subsequent days. The charges were that the placenta not having been expelled within an hour after birth, and both mother and child being ill, she did not send for a doctor; that on the arrival of Dr. Pershouse on September 22 she did not carry out his instructions, declining to attend the patient or to administer a vaginal douche, and subsequently on the 23rd and 24th administering what purported to be vaginal douches per rectum; and that she fed the child on oatmeal and gin, thereby conducing to its death on September 24. She was also charged with being generally uncleanly, with not taking appliances and antiseptics, and with not keeping a register of cases. Dr. Pershouse said he was called by the mother-in-law of the patient five days after the birth of the child, though the midwife objected to his visiting the case. He found all the symptoms of puerperal fever; the midwife refused to come and administer the vaginal douches he prescribed, and when subsequently she administered them it was per rectum. This he was told by the patient. On the 24th Dr. Pershouse and his partner removed a portion of the placenta from the patient. He saw the baby on the same day, and found it dying, and was told it had been fed on oatmeal and gin. The baby died a few hours later. Dr. Thresh, Medical Officer of Health for the county of Essex, said he saw the woman on September 17, and found her unc'ean, having no bag, book, or appliances. She admitted to him all the charges made against her by Dr. Pershouse. She belonged to the Peculiar People, and objected to calling in a doctor. It was unanimously decided to remove her from the roll.

Charges were then brought against Elizabeth Jacklin, to the effect that she was of intemperate habits, and was in particular intoxicated on August 2, 1905, in her own home; that she was under the influence of drink on June 23, 1905, at the offices of the Medical Officer of Health; that she was habitually uncleanly, did not take appliances to a confinement, and did not keep a register of cases. Miss Bowers, Sanitary Inspector for Nottingham and Acting Inspector of Midwives, said she saw the woman eight times in her own home, and on each occasion found her not sober; she was present at the meeting at the office of the Medical Officer of Health, and testified that she appeared under the influence of drink at the time. She had practically no appliances, and did not get them, though she was repeatedly warned to do so. She said she had one washable dress, and afterwards denied having any. Her name was removed from the roll.

Esther Smith was charged with being habitually unclean, with not taking appliances to confinements, with not disinfecting her hands before touching the genital organs, and not washing patients' external parts with soap and water and an antiseptic solution before making the first internal examination. Evidence was submitted by the Inspector of Midwives, who said she had no appliances and antiseptics, no washable bodice, and was uncleanly. The visitor of the Darlington Sick Nursing Society gave long detailed accounts of six cases in which Esther Smith was said to have been guilty of most unpardonable negligence, on the authority of various doctors. The woman denied the greater part of the charges. Her name was removed from the roll.

The case of Mary Ann George, of Worcester, again came up for consideration. She was convicted of felony on January 27, and on October 27 was fined £1 for not having notified the fact of her intention to practise to the local supervising authority. She was also charged with not taking appliances and antiseptics to confinements, and with not keeping a register of cases. Her name was removed from the roll. In view of the fact that a clergyman had given the woman a certificate of good character, which enabled her to be enrolled, knowing as he did that she had been convicted of felony, the Board decided to send the papers dealing with this part of the case to the Public Prosecutor.

### Practical Hints.

**We welcome notes on practical points from nurses.**

### BATHS.

The subject of "baths" enters largely into a nurse's training, and a few directions for the administration of the simpler forms will not, I hope, be out of place.

The first thing to be done, as all of you probably know, with a new patient is to take her temperature, pulse, and respiration, and, if in a fit condition, she is taken to the bath-room and a hot bath given. The nurse should carefully examine the body to see if there are any sores, bruises, etc., or anything abnormal on it, and report such to the Sister. Most hospital patients are not remarkable for their cleanliness on admission, and I have always found it best to half fill the bath, so that the body is well covered, and the water at a temperature of 100° to 110°. Use plenty of soft soap and, if necessary, turpentine, for the feet and hands, taking care that this is washed off with soap and water. In the case of women the head must be well lathered with soap and afterwards dried with hot towels. It is a good plan to lay the long hair over a hot-water bottle to further dry it, after the patient is in bed. The nurse must in all cases have everything she is likely to require ready before beginning to bath her patient, who must not be left alone in the bath-room or bath, as many patients are subject to attacks of faintness. These are more likely to occur after a sudden change from the cold outer atmosphere to the warmth of a bath-room.

In bathing children, especially, be careful to put the cold water into the bath first, and so prevent any risk of the bottom of the bath becoming overheated. A child must be put in gradually, so as not to frighten it.

Cold baths may be ordered in the case of hyperpyrexia. These must only be given by medical direction, as they are necessarily attended by a certain amount of shock to a patient who has had a long-continued temperature of 104° or 105°. Strip the patient in bed, lay a bath towel over her, remove the draw-mackintosh, and, with help, gradually lower her, by means of the long under sheet, into the water. This should be 90°, and must cover her well; add cold water by degrees till the thermometer registers 70° or 65°. The patient's temperature should be taken frequently in the rectum or mouth while she is in the bath, and she should be taken out before it falls below 99°; otherwise she may become collapsed, and more harm than good will be done. It is often well to give some stimulant while the patient is in the bath—hot coffee, brandy, or beef tea. The bed should be prepared with a large receiving mackintosh, so that she can be lifted out in the same way and placed on the bed, carefully dried, mackintosh and wet sheet removed together, a warm nightdress put on, and a hot-water bottle to the feet. If there are any signs of shivering put a hot blanket next her and a couple of hot-water bottles at either side. These should be taken away as soon as she recovers. In twenty minutes the temperature must be again taken. Another way of reducing temperature is by tepid sponging. Let your patient lie on a blanket and mackintosh, place a hot-water bottle at the feet, and sponge slowly from the chest downwards with water at a temperature of 90°, paying special attention to the axillæ, arms, and inside of thighs. As the water becomes warm by the constant dipping of the sponge heated by contact with the body, it is as well to have a jug of cold water handy, and add a little from time to time. Turn the patient on her side and continue the sponging over the back and legs—particularly down the spine. Cover her lightly for twenty minutes; and if little reduction is made in the temperature by that time, sponge again in the same manner.

Hot-air baths are frequently ordered in cases of Bright's disease to promote perspiration.

The patient who is usually confined to bed should be stripped and lightly covered with a thin blanket, a mackintosh and blanket placed under her, a large body cradle on the bed over the patient, and on this should be hung a thermometer. Cover all with a couple of large mackintoshes and three thick blankets, tuck well in, especially at the neck, leaving only a small aperture at one side or at the foot for the funnel of the lamp, which should be above the patient's body. Light the spirit lamp and stand it on a metal plate or tray below the funnel. Some lamps give out more heat than others, but as a rule thirty to forty minutes should bring the temperature inside the cradle up to 120° or 140°. A good plan is to give the patient a drink of hot barley-water or lemonade directly she begins to be hot, this helps to make her perspire freely. As soon as she does so put out the lamp and draw out, without exposing her to the air, the cradle and mackintoshes and let her lie for an hour or more closely covered by blankets. While the patient is in the bath a careful watch must be kept on the pulse and the bath must be stopped if she complains of feeling faint.

Vapour baths are given in much the same way, but a steam kettle is used in place of dry hot air.

Carbonate of soda baths I have found infinitely soothing in the case of body burns among children. About two ounces to an ordinary sized child's bath. Place the bath before the fire and gently immerse the child in the water at about blood heat, gradually adding hot water as she can bear it. When the clothes are still on remove only the outer ones and cut away the inner ones in the water. If brandy is ordered it may be given while the child is in the bath if she can swallow, or per rectum by means of a tube and funnel. These baths are a great help in recovering from shock, and the child may remain in them for some hours if the water is kept hot and a blanket thrown over all.

### Everybody's Opinion.

[Correspondence on all subjects is invited, but we cannot in any way be responsible for the opinions expressed by our correspondents. No communication can be entertained if the name and address of the correspondent are not given as a guarantee of good faith, but not necessarily for publication. All correspondents should write on one side of the paper only.]

### A WARNING TO NURSES.

"A Former Sister" at Winchester writes : In thanking "Sister B." for exposing the roguery of the man mentioned in your issue of last week, I should like to add that when he was first admitted to Winchester Hospital he gave his name as "Neville," and described himself as an actor "down on his luck" through constant attacks of malaria. He also said he belonged to an aristocratic family, but was disowned as the "black sheep" through his own fault. He quietly asked me for the loan of £1 to be paid with interest, but as I had doubts about him I declined to accede to the request. I also suspected him as having stolen a purse of money belonging to a fellow-patient. Being a good looking man, he tried to make love to nurses without success. I sincerely hope that his career is now checked. On his return to Winchester he tried the same tactics.

W. Hudson Marsden, L.R.C.P. and M.R.C.S., Assistant House Physician of the Royal Devon and Exeter Hospital, Exeter, writes : As my attention has been drawn to a letter by "Sister B." of the York County Hospital, appearing in your paper, I wish to thank you and her for giving publicity to the wiles of that impostor who has been personating me and other medical men in such a scandalous manner. I recognise easily in her description a man who came here and was admitted on August 5, 1904, giving the names of Charles Vernon Heathcote, and his address as Folkestone, in Kent. He had the same catalogue of symptoms and behaved in much the same way as "Sister B." describes. He went out on October 7, 1904, and sent a telegram from Bristol under the name of "McGrath" stating that Heathcote had shot himself. He went to the hospital at Bath and performed the same antics there. The next I knew was that I am supposed to be in such indigent circumstances as to apply for admission as a patient to a public institution. I am very glad that "Sister B." has issued her warning, and in emphasising that warning I hope that all hospitals will close their doors to such an individual and refer him to other quarters more suitable for such as he. He has made himself so notorious in this section of England that there awaits for him a very cordial reception should he ever appear here again.

### NURSES AND INTEMPERANCE.

"A Sorrowful Witness" writes from 168 Edmund Street, Birmingham : I quite agree with your comments upon the way in which nurses are tempted to drink in some families in which they are ministering. There is, however, another side to the question. I am intimately related to a family of abstainers—who were, indeed, among the first pioneers of the movement. A daughter of that family married an abstainer, and lived to be 45 years of age without ever taking intoxicants. She had a serious illness, and a nurse had to be engaged for several weeks. The nurse was herself a spirit-drinker, and induced her patient to drink, and to believe it necessary. The patient emerged from her illness as a habitual drunkard. I have had her under my own roof for weeks in the endeavour to save her, but all efforts have proved vain. She has been a sly drunkard for at least ten years now, and I fear will be so until the sad end comes. I am thankful that her father and mother never lived to see her the victim of the drink against which they so long battled while they lived. I enclose my card.

### THE GENERAL MEETING OF THE ROYAL BRITISH NURSES' ASSOCIATION.

Miss Helen Todd, Matron of the Royal National Sanatorium, writes : I have had my attention drawn to the report of the general meeting of the Royal British Nurses' Association held on January 17 and published by you on January 27. In it you mention my name, and even report words that I am supposed to have said at the meeting. I am not a member of the Royal British Nurses' Association, nor have I ever attended any of their meetings, much less spoken at one. I must say I should have dearly liked to have been present on the 17th, and to have added a vote to those recorded for a fair representation of nurses on their own governing body, but not being a member of the Association I fully realised that I was not qualified to do so. May I ask you to be good enough to insert this contradiction in your next issue?

[We regret that in the report supplied to us the remarks made by another lady were wrongly attributed to Miss H. Todd.—Ed. The Hospital.]

### MATRONS AND INTENDING PROBATIONERS.

"Liscard" writes : I see in your paper a certain hospital advertising for probationers. I should very much like, if possible, to have your opinion upon the way I was treated in connection with that hospital. I wrote to the Matron, and she sent me an application form. It stated that a premium was required, so I wrote and said I understood that they were abolishing premiums. I then had a reply, saying that that was so, and if I would send in my form I should come under that rule. I did so, sending as my references several very well-known people who had known me nearly all my life. These friends received forms with a great many questions, which they promptly answered and returned. A few days later I had a letter saying that the matron, before accepting me as a probationer, would like to see me. The form had not stated that a personal interview was necessary, and it was a very long way, but I decided to go, as I was very anxious to get into hospital. I left home at six o'clock in the morning, arriving at two o'clock, and went straight to the hospital, as I did not know anyone in the town. I saw the matron, and she just asked me one or two questions, one of which was, "Was I willing to pay a premium?" although I had stated quite clearly in my letter that I was not. I again said that I was not. I was not asked to wait there for the next train, although the matron knew I was an absolute stranger to the place; but I went to the station and waited there until a quarter to five o'clock, when I took the train, arriving home after eleven at night. A day or two later I had an intimation from the matron saying that "On consideration she could not accept me as a probationer in her hospital." There was no reason whatever stated, and I think that for the sake of others who might do the same thing, the matter should be known. The fare cost me over £2, which would be a considerable amount to most women.

### THE CHARITABLE NURSE.

"G. C." writes : I feel compelled on behalf of my fellow-workers to give my opinion as to the cause of a good deal of the lack of charity which exists amongst a profession where ample scope is given for all that is best in woman, both intellectually and morally, and I am afraid the fault will not be overcome while the cause still remains. "Charity suffereth long and is kind," but, alas! nowadays how little is shown or given on either side! The lack of charity is felt soon after a girl enters upon her career. In a good many instances we find the matron or sisters in the institution showing favouritism to some, whilst treating others indifferently, and naturally these distinctions give rise to feelings of jealousy, and thus strife and mischief are caused. Hence it is indeed hard for a girl to go through her probation and remain unblemished and possessed of charitable feelings towards all. Nevertheless, there are women, and many of them, possessing the qualities which combine together to make a true nurse, namely, ready hands, kind hearts, and observant eyes, who emerge from their training fully possessed with the milk of human kindness. Now comes the trial. If the nurse has chosen private nursing, it will indeed be found increasingly hard to proceed and yet remain charitable to one and all. Patients and their friends, I am afraid, often treat us as if we required neither rest, sleep, nor recreation. They fail to remember that even if a nurse has the best will in the world she cannot give her full powers to her patient if she neglects herself, and when we are compelled to remonstrate we are deemed creatures without feeling and without charity, though, perhaps, in reality we have been devotion itself to our patient, sparing nothing in striving to alleviate his sufferings. There is another cause. If a household dreads the advent of a nurse, well, when such a needful accessory does arrive upon the scene, one can imagine that she would not be welcomed in a charitable spirit. Her natural right, coming to help as she does, causes her to establish a personal relationship between herself and her patient, and, if the friends would not misjudge her motives, in nine cases out of ten they would find her a comfort and a blessing in time of sickness instead of a source of irritation. If every woman who took up nursing would put her soul into her work she would find little time to indulge in uncharitable and ill-natured talk concerning others.

### Appointments.

[No charge is made for announcements under this head, and we are always glad to receive and publish appointments. The information, to insure accuracy, should be sent from the nurses themselves, and we cannot undertake to correct official announcements which may happen to be inaccurate. It is essential that in all cases the school of training should be given.]

Ashton-under Lyne District Infirmary.—Miss Worthington has been appointed sister. She was trained at Guy's Hospital, London, and has since been sister at the Children's Hospital, Bradford.

Beckett Hospital, Barnsley.—Miss A. B. Crawley has been appointed sister. She was trained at Leeds General Infirmary, and has since been on the staff of the Cancer Hospital, London. She has also done private nursing at Leeds.

Charing Cross Hospital, London.—Miss Agnes McBrick-Haugh has been appointed sister to the Golding Ward, and Miss Grace Shaw has been appointed theatre sister. They were both trained at Charing Cross Hospital.

Devonport Isolation Hospital.—Miss Maud Ashworth and Miss Grace Kerr Bell have been appointed nurses. Miss Ashworth was trained at the County Hospital, Leinster, and the Fountain Fever Hospital, Tooting, London. Miss Kerr Bell was trained at the Edinburgh City Fever Hospital.

Downs School, Banstead Road, Sutton, Surrey.—Miss M. V. Ryott has been appointed house matron. She was trained at the Royal Infirmary, Bradford, and has since been sister at the Royal County Hospital, Ryde, and sister and night superintendent at the Southwark Infirmary, East Dulwich.

General Hospital, Birmingham.—Miss Mildred A. Nodal has been appointed assistant matron. She was trained at the London Hospital, where she was afterwards massage sister. Subsequently she was ward sister and assistant matron at the National Hospital for the Paralysed and Epileptic, Queen Square, London. She has also had experience at the Royal London Ophthalmic Hospital, Moorfields, London, and at the London General Lying-in Hospital, and has acted as temporary night superintendent at the Shoreditch Infirmary.

Manchester Southern Hospital.—Mrs. A. E. Firth has been appointed matron and Miss Kate Taylor has been appointed sister. Mrs. Firth was trained at the Stanley Hospital, Liverpool, and has since been sister at the Jessop Hospital for Women, Sheffield, and sister at the Manchester Southern Hospital. She has also done private nursing, and holds the certificate of the Central Midwives Board. Miss Taylor was trained at the Queen's Hospital, Birmingham. She has since been private nurse in connection with the Leeds City Hospital and the Liverpool City Hospital. She has also been nurse matron at Foxall, near Burton, and sister at Tendray Hospital, Barnsley.

Norfolk and Norwich Hospital.—Miss Edith Grocott has been appointed night superintendent. She was trained at the Royal Infirmary, Liverpool, where she was subsequently private nurse in connection with that institution. Since then she has been sister at the Northampton General Hospital.

Norfolk and Norwich Hospital, Norwich.—Miss L. Shepherd has been appointed housekeeper. She was trained at Leeds Infirmary, and has since been sister at the Chesterfield and North Derbyshire Hospital.

Northampton General Hospital.—Miss F. M. Borton has been appointed sister. She was trained at the Westminster Hospital, London, and has also been on the private staff of that institution.

Nottingham Workhouse Infirmary.—Miss Mary Coyne and Miss Emma Elizabeth Shepherd have been appointed charge nurses. Miss Coyne was trained at Prescot Poor-law Infirmary, and has since been charge nurse at Aston Poor-law Infirmary. Miss Shepherd was trained at Leeds Workhouse Infirmary, and has since been ward sister. She was previously matron's assistant at Bromsgrove Workhouse.

Sheffield Lodge Moor Hospital.—Miss E. H. Lapham has been appointed sister. She was trained at the Meath Hospital and County Dublin Infirmary, and has since been charge nurse at the Isolation Hospital, Norwich, and night nurse at the Cottage Hospital, East Cowes.

Sussex County Hospital, Brighton.—Miss Ida Kathleen Szezepankska has been appointed assistant matron. She was trained at the North-Eastern Hospital for Children and King's College Hospital, London. She has been temporary assistant matron at Nottingham General Hospital, and a sister of the Army Nursing Reserve Service. She holds certificates for massage and electricity, and for midwifery.

Woolwich Infirmary.—Miss Ellen M. Denyer has been appointed ward sister. She was trained at St. Olave's Infirmary, Rotherhithe, S.E., and has since been staff nurse at the Cottage Hospital, Redhill, Surrey, and staff nurse at Camberwell Infirmary.

### "The Hospital" Convalescent Fund.

The Hon. Secretary acknowledges with grateful thanks the receipt of 1s. from Miss Butcher, per the Royal National Pension Fund; and 5s. from "Alison," being 2s. 6d. as her annual subscription, and 2s. 6d. as a donation.

### Notes and Queries.

**REGULATIONS.**

**The Editor is always willing to answer in this column, without any fee, all reasonable questions, as soon as possible.**

**But the following rules must be carefully observed.**

**1. Every communication must be accompanied by the name and address of the writer.**

**2. The question must always bear upon nursing, directly or indirectly.**

**If an answer is required by letter a fee of half-a-crown must be enclosed with the note containing the inquiry.**

*Convalescent Home for Nurse.*

(133) Can you tell me of a Convalescent Home near Lancaster where a nurse could go. She is practically without means and recovering from illness?—*Nurse Violet.*

If not too far, write to Miss Shaw, Matron, Parkwood, Henley-on-Thames. Here trained nurses are received free. Many convalescent homes are closed just now. There is a Home for Gentlewomen at 42 Park Road, Southport, 10s. and 12s. a week, open all the year round. Also at New Brighton, Cheshire, there is a Convalescent Home for Women and Children, 6s. 6d. to 21s. weekly, and the Royal Alexandra Convalescent Home at Rhyl, North Wales, 12s. and 20s. weekly.

*Nurse Going out to Africa.*

(134) Can I, as a nurse (Midwife and L.O.S.), get a reduction on my fare to Africa, where I hope to go in May?—*S. F.*

Possibly the South African Colonisation Society, 47 Victoria Street, Westminster, or the British Women's Emigration Society, The Imperial Institute, S.W., might assist you; but there are so many nurses now in South Africa that it is very doubtful. Your best plan otherwise would be to advertise.

*Want of Work.*

(135) Can you help me? I am 59 years of age, and now that all young nurses are required, I can get no work. I would set up a small business if I had the capital.—*Yorkshire Nurse.*

(136) I am a trained nurse, aged 46, and find it impossible to get regular work. Can you tell me anyone I could apply to?—*Irish Nurse.*

We regret that we do not know of any fund or agency that would be of service in these unfortunate cases.

*National Health Society.*

(137) Please will you give me the address of the National Health Society?—*C. D.*

53 Berners Street, London, W.

*District Nursing.*

(138) Will you kindly inform me of a good district home for trained nurses to get knowledge of district nursing, or where I could get to know of such to go to for a short period?—*A. B.*

Apply to Queen Victoria Jubilee Nursing Institution, 120 Victoria Street, S.W., or to the Maternity and District Nurses' Home, Howards Road, Plaistow, E. Otherwise advertise in the "Nursing Mirror."

*Feeble-minded.*

(139) Is there any home where a woman, thirty-three years of age, suffering from chronic Bright's disease, and also a little simple-minded, could be admitted free? She has been a servant. I have written to some Incurable Homes but they say she is not a suitable case. She is quite able to do housework or light needlework. Are there any homes for feeble-minded women not insane or idiots?—*Nurse O.*

Write to the National Association for Promoting the Welfare of the Feeble-minded, 53 Victoria Street, S.W.

*Salt.*

(140) Do you consider salt a necessity, and can it act on the system and so prevent the disfiguring "acne" so often seen in young girls?—*Sister Nancie.*

Salt is necessary for the maintenance of life. There are several causes of acne, and it is not possible, therefore, to answer your second question without a personal knowledge of the case.

**Handbooks for Nurses.**

| | Post Free. |
|---|---|
| "How to Become a Nurse: How and Where to Train." | 2s. 4d. |
| "Nursing: its Theory and Practice." (Lewis.) ... | 3s. 6d. |
| "Nurses' Pronouncing Dictionary of Medical Terms." | 2s. 6d. |
| "Complete Handbook of Midwifery." (Watson.) ... | 6s. 4d. |
| "Preparation for Operation in Private Houses." ... | 0s. 6d. |

Of all booksellers or of the Scientific Press, Limited, 28 & 29 Southampton Street, Strand, London, W.C.

### For Reading to the Sick.

"LIGHT OF OUR LIFE."

O Thou One
Light of our life, in these dark later days,
When earth-born mists and storm-clouds veil the Sun,
Grant us to know Thee when Thy first bright rays
Pierce the soul's gloom, and afterward Thy praise
Tell, nothing doubting, till our years be done.

*E. H. Day.*

A firm belief in His Providence, His Goodness and Wisdom, which suit every trial to the sufferer's need, is a great help towards patient endurance. If sharp sickness be laid on you, it may be that you did not use the gift of health wisely, or to God's Glory; perhaps you abused it in seeking mere earthly enjoyment, and God in His Love calls you apart from the world, to open your eyes to your soul's need: He lays a chastening Hand upon your body, but while He tries it with pain and distress, He will not fail to soothe and strengthen it as is best for you.

Those who can say, "It is my Father's Will," are assuredly learning to "use the vale of misery as a well"; their "pools are filled" with water of consolation and refreshment. Be of good cheer—God has not many willing victims. Do you strive to be one of the few, and nothing will overcome you; "Let them which suffer according to the Will of God commit their souls unto Him in well-doing, as to a faithful Creator."

Learn to say, "I know, O Lord, that Thy judgments are right, and that Thou of very faithfulness hast caused me to be troubled." I would not avert one blow Thy Mercy wills to deal me, however hard it be. Impatience and fretting under trial does but increase our suffering, whereas such meek submission sanctifies all suffering, and fills the tortured heart with peace amid its anguish.

If Jesus Himself was a meet Sacrifice to the Father's Will, what are we poor earthly creatures that we should murmur, or grudge our little offering of health, of all even that makes life sweet to us, of that very life itself? Let His Holy Will work in you, whatever it may cost you; let it reign supreme, and do you strive to offer yourself and all you have as a willing and acceptable sacrifice to it. Worship the Hand Which smites, with Job, who "fell down and worshipped, saying: the Lord gave, and the Lord hath taken away, blessed be the Name of the Lord."

*Guillore.*

Be sure that your mere silent willing endurance is a true act of adoration; and thus, come what may, weariness, pain, desolation, destitution, loneliness, all will carry on His gracious work in you, and amid the sharpest pressure of suffering you will be sending up to His eternal throne the precious incense of submission and trust. "Though He slay me, yet will I trust in Him."

And though beneath His Cross the sword
Of sorrow pierced each loyal heart,
They most who sorrow with their Lord
In Resurrection joy have part.

*E. H. Day.*

## Figures and Tables

**Fig. 3. f1:**
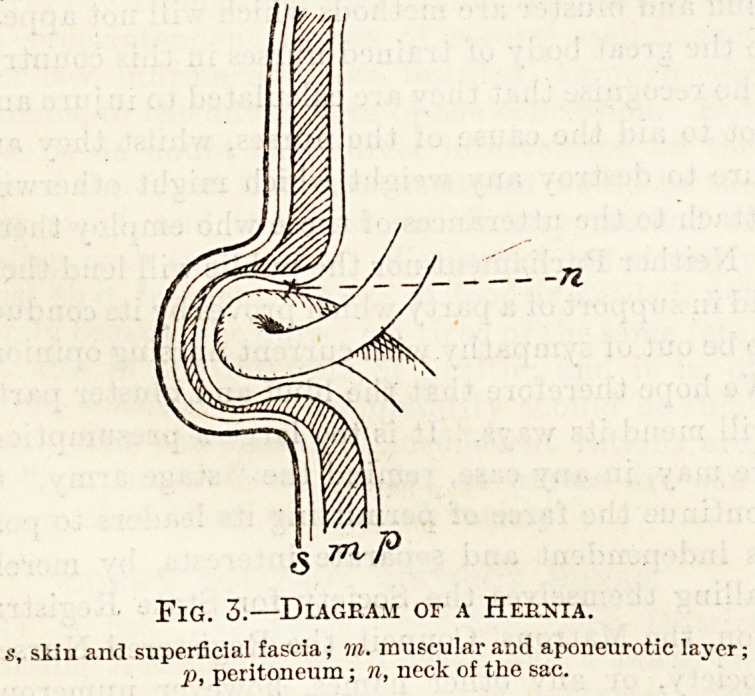


**Fig. 4. f2:**